# Supplementary material for: Stealth Liposomes Encapsulating a Potent ACAT1/SOAT1 Inhibitor F12511: Pharmacokinetic, Biodistribution, and Toxicity Studies in Wild-Type Mice and Efficacy Studies in Triple Transgenic Alzheimer’s Disease Mice
Source: Int J Mol Sci. 2023 Jul 2;24(13):11013. doi: 10.3390/ijms241311013 (PMC10341764; doi:10.3390/ijms241311013)
Supplement: Supplementary file 1 [file ijms-24-11013-s001.zip › ijms-2412717-supplementary.pdf]

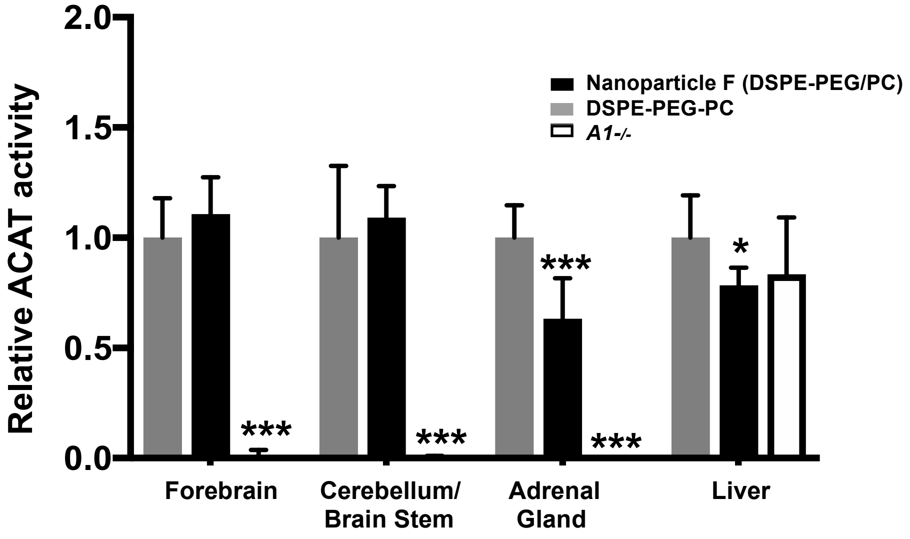

Figure S1. **The oral gavage was performed to 8 WT male mice, at age 4-months old**. Four mice were injected with nanoparticle alone, and the other four were injected with nanoparticle F. One *Acat1*$^{-/-}$ mouse served as the control. This study involved daily oral gavage administration of nanoparticle with or without F for 4 consecutive days, and measured ACAT activity 6 h after the final administration. Mice were sacrificed, ACAT enzyme activities were measured in forebrains, cerebellums/brain stems, adrenal glands, and livers isolated from the individual mouse. $p < 0.001$ ***, $p < 0.05$ *.

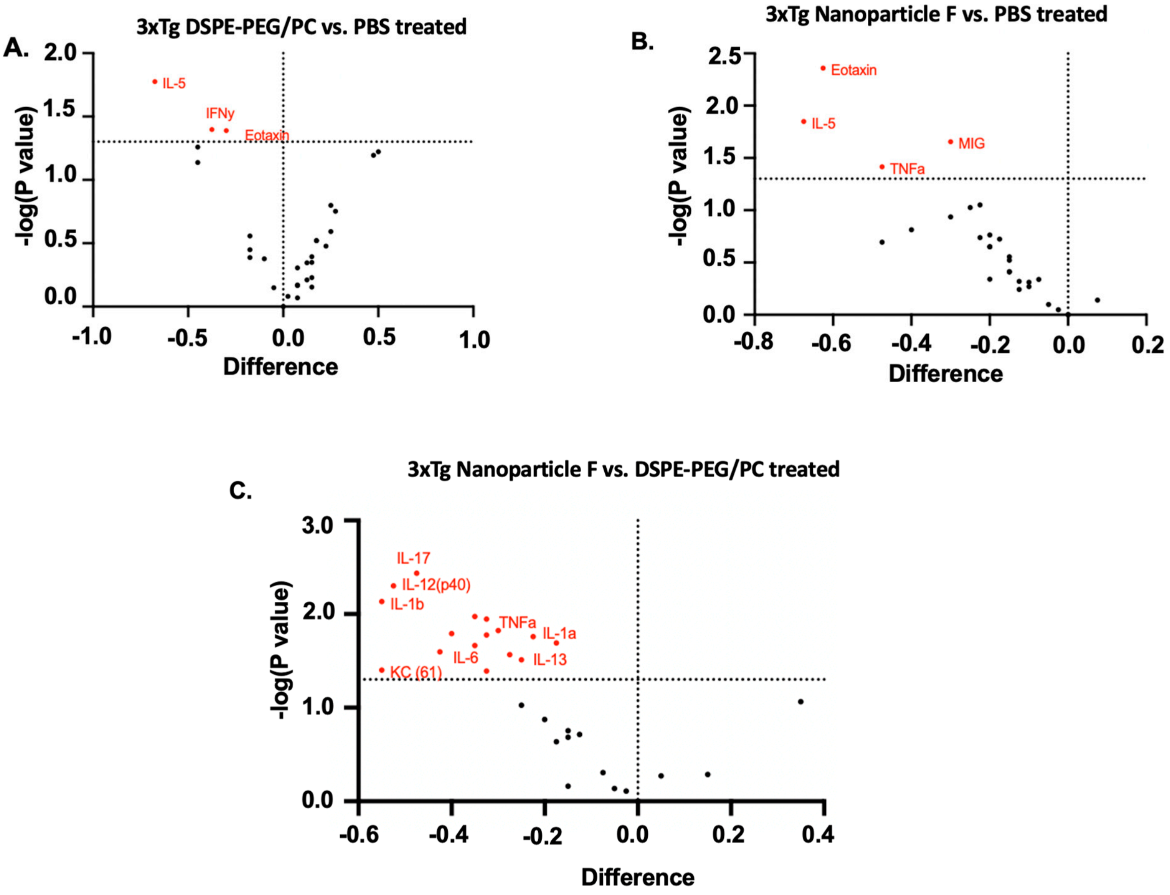

Figure S2. Brain cytokines differentiate upon treatment with DSPE-PEG/PC and nanoparticle F. Volcano plot showing cytokines change upon (A) DSPE-PEG/PC treatment vs. PBS. (B) nanoparticle F vs. PBS. (C) nanoparticle F vs. DSPE-PEG/PC in 3xTg mice. Horizontal dashed line is determined at p = 0.05. Data points above the dash line are statically different at $p < 0.05$ determined by unpaired student t-test. N = 4 mice/group.
